# RACISM AND DISCRIMINATION EXPERIENCES AMONG STAFF IN LONG TERM CARE HOMES

**DOI:** 10.1093/geroni/igad104.3553

**Published:** 2023-12-21

**Authors:** Liwam Nerayo, Jasmine Travers, Laura Wagner

**Affiliations:** University of California, San Francisco, San Francisco, California, United States; New York University, New York City, New York, United States; University of California, San Francisco, San Francisco, California, United States

## Abstract

In long term care facilities (LTCFs), there is a growing population of people with dementia and an increasingly racially and ethnically minoritized care workforce. Previous research has shown that relationships between residents with dementia and minoritized care workers have been contentious, involving several racial and discriminatory acts (e.g., racial slurs, physical abuse). This has led to an unsupported and burnt out care workforce. The aim of this study is to develop a survey tool, aimed at examining experiences and understanding the prevalence of racism and discrimination in the LTC care workforce. We pilot tested the study in two LTCFs and performed content validity with experts. We then deployed the survey across 560 facilities in racially and ethnically diverse states. When analyzing responses from the first 113 nursing assistants and vocational nurses, we found that many of the respondents reported having positive perceptions of residents with dementia and no experiences with racism. However, among the one-third of care workers who did experience racism, we discovered that respondents primarily experienced racism through verbal insults (48%) and care refusal (23%). Over 87% of victims of this experience felt supported by their manager, yet only 50% reported getting reassigned. Finally, almost 80% responded that it is more challenging to care for residents who exhibit racist behavior. This feedback on the perspectives and experiences of the care workers who did experience racism can inform improvements for dementia care training and offer discussions among stakeholders and families. Additional data from more diverse LTCFs is forthcoming.

